# Medical Legislation—Professional Honors

**Published:** 1877-08

**Authors:** 


### Editorial.

### Medical Legislation—Professional Honors.

Medical societies have generally held their annual meetings, the characteristic feature being prominence of the *legislative section.* Now it may be well enough for the American medical associations to pass a resolution, giving sanction of law to the prevailing custom of an ex-president acting as nominating committee, selecting the victims upon whom the honors of the association are to be unanimously conferred. In this connection it may be allowable to suggest that professional attainment, distinguished service, or other merit is not to be considered, residence in New Orleans, Boston, San Francisco, Charlestown, New York, or Philadelphia determining the choice in connection with political partizanship. These great centers of political influence are not to be forgotten by the ex-president composing the nominating committee; sectional and political considerations must be allowed full control, in matters of professional progress, and scientific attainment. The honors of our noble profession are not to be thrown away upon the hard working, progressive, intelligent, honest and trustworthy members, regardless of sectional distinctions. (?)

